# Long-term fasting improves lipoprotein-associated atherogenic risk in humans

**DOI:** 10.1007/s00394-021-02578-0

**Published:** 2021-05-07

**Authors:** Franziska Grundler, Dietmar Plonné, Robin Mesnage, Diethard Müller, Cesare R. Sirtori, Massimiliano Ruscica, Françoise Wilhelmi de Toledo

**Affiliations:** 1 Buchinger Wilhelmi Clinic, Wilhelm-Beck-Straße 27, 88662 Überlingen, Germany; 2 Charité-Universitätsmedizin Berlin, corporate member of Freie Universität Berlin, Humboldt-Universität zu Berlin, and Berlin Institut of Health, Berlin, Germany; 3 MVZ Humangenetik Ulm, Karlstraße 31-33, 89073 Ulm, Germany; 4 Gene Expression and Therapy Group, Faculty of Life Sciences and Medicine, Department of Medical and Molecular Genetics, King’s College London, Guy’s Hospital, 8th Floor, Tower Wing, Great Maze Pond, London, SE1 9RT UK; 5 MVZ Labor Ravensburg, Elisabethenstraße 11, 88212 Ravensburg, Germany; 6 Department of Pharmacological and Biomolecular Sciences, Università Degli Studi di Milano, Milan, Italy

**Keywords:** Buchinger Wilhelmi fasting program, Long-term fasting, LDL subclasses, HDL subclasses, Lipoproteins

## Abstract

**Purpose:**

Dyslipidemia is a major health concern associated with an increased risk of cardiovascular mortality. Long-term fasting (LF) has been shown to improve plasma lipid profile. We performed an in-depth investigation of lipoprotein composition.

**Methods:**

This observational study included 40 volunteers (50% men, aged 32–65 years), who underwent a medically supervised fast of 14 days (250 kcal/day). Changes in lipid and lipoprotein levels, as well as in lipoprotein subclasses and particles, were measured by ultracentrifugation and nuclear magnetic resonance (NMR) at baseline, and after 7 and 14 fasting days.

**Results:**

The largest changes were found after 14 fasting days. There were significant reductions in triglycerides (TG, − 0.35 ± 0.1 mmol/L), very low-density lipoprotein (VLDL)-TG (− 0.46 ± 0.08 mmol/L), VLDL-cholesterol (VLDL-C, − 0.16 ± 0.03 mmol/L) and low-density lipoprotein (LDL)-C (− 0.72 ± 0.14 mmol/L). Analysis of LDL subclasses showed a significant decrease in LDL1-C (− 0.16 ± 0.05 mmol/L), LDL2-C (− 0.30 ± 0.06 mmol/L) and LDL3-C (− 0.27 ± 0.05 mmol/L). NMR spectroscopy showed a significant reduction in large VLDL particles (− 5.18 ± 1.26 nmol/L), as well as large (− 244.13 ± 39.45 nmol/L) and small LDL particles (− 38.45 ± 44.04 nmol/L). A significant decrease in high-density lipoprotein (HDL)-C (− 0.16 ± 0.04 mmol/L) was observed. By contrast, the concentration in large HDL particles was significantly raised. Apolipoprotein A1 decreased significantly whereas apolipoprotein B, lipoprotein(a), fibrinogen and high-sensitivity C-reactive protein were unchanged.

**Conclusion:**

Our results suggest that LF improves lipoprotein levels and lipoprotein subclasses and ameliorates the lipoprotein-associated atherogenic risk profile, suggesting a reduction in the cardiovascular risk linked to dyslipidemia.

**Trial Registration:**

Study registration number: DRKS-ID: DRKS00010111 Date of registration: 03/06/2016 “retrospectively registered”.

**Supplementary Information:**

The online version contains supplementary material available at 10.1007/s00394-021-02578-0.

## Introduction

The voluntary abstinence or strong limitation of caloric intake for a limited period of time, also known as fasting, has gained popularity in the last decade to prevent and treat age-related diseases. Long-term fasting (LF) of 2 to 21 days or more [1], intermittent fasting (IF) [2], and nutritional strategies like calorie restriction, have been shown to reduce risk factors for cardiovascular disease (CVD), such as dyslipidemia, body mass index (BMI), abdominal circumference, hypertension, and insulin resistance [3–6]. LF with a supplementation of 250 kcal per day is well tolerated physically and emotionally [1]. During LF, a metabolic switch from the use of food-derived glucose to lipids and ketones (G-to-K) occurs 10–16 h after the last meal [7]. This leads to global biochemical changes in lipid metabolism with known health benefits [7]. Earlier studies indicated a reduction in total cholesterol (TC), triglycerides (TG), low-density lipoprotein cholesterol (LDL-C), and to a lesser extent, a rise of high-density lipoprotein cholesterol (HDL-C) [8]. However, lipoprotein composition was never investigated in detail. Studies on alternate-day fasting in obese and non-obese subjects reported modest TC, LDL-C, and TG reductions, with a modest shift towards larger LDL particles and a reduction in small-sized LDL [9]. In metabolic syndrome patients, IF with a 10-h time-restricted eating led to decrease the atherogenic profile [10]. Other dietary approaches like the Dietary Approach to Stop Hypertension (DASH) and Mediterranean diets have shown comparable results [11, 12]

A rapid decrease of TG is a main feature of fasting in humans after cessation of food intake [8]. Since the fasting protocol used in the present study provides a supplementation of 250 kcal containing zero fat, we hypothesised that chylomicrons disappear and that a massive remodelling in lipoproteins is triggered [8]. In this scenario, both epidemiological and genetic studies have reported the possible role of TG as a causal risk factor in atherosclerotic cardiovascular disease (ASCVD) [13]. Very low-density lipoprotein (VLDL) particles are heterogeneous in composition and size, the larger being VLDL1 particles (enriched in TG) and the smaller VLDL2 particles (containing less TG) [14]. Lipoprotein lipase acts on VLDL to hydrolyse core TG and convert particles to LDL (via IDL) as the terminal product of lipolysis [15]. Cleavage of TG by lipoprotein lipase (LPL) liberates free fatty acids, partly converted in the liver to the ketones β-hydroxybutyrate and acetoacetate [16]. Ketone bodies are an important source of energy for most body cells during fasting [1, 8, 17], leading to a fully compensated acidosis, and exerting an anorectic activity [18].

Low carbohydrate/high-fat restrictive nutritional strategies improve atherogenic dyslipidemia in metabolic syndrome patients regardless of weight loss [19] suggesting that, in analogy, fasting metabolism, relying essentially on endogenous lipids, could have a comparable effect. LDL is considered to be the major and causal driving force in the development of ASCVD [20]. LDL particles may be produced via two pathways, either by extensive intravascular remodelling of hepatic VLDL or to a lesser extent by de novo liver secretion. The former pathway typically predominates [21]. LDL are a continuum of particles distributed over densities ranging from 1.019 to 1.063 g/ml, further sub-classified into large buoyant LDL1, intermediate LDL2 and small dense LDL3 (sdLDL) [22]. The sdLDL particles are characterized by a low lipid/protein ratio and small particle size, both favouring an enhanced transcytosis across the arterial endothelium triggering atheroma formation [23, 24]. HDL changes are of special interest, since HDL are recognized as a protective factor against arterial disease. Small variations in their levels may lead to a change in risk [25].

The aim of the present study was to document how LF, carried out according to the program of the Buchinger Wilhelmi clinics (BWC), changes lipoprotein subclass distribution in humans. For this purpose, two methods were applied to analyze lipoprotein subclasses: the separation of lipoprotein subclasses by density gradient ultracentrifugation, and the measurement of lipoprotein particle concentration and particle size by NMR spectroscopy. The protocol includes a 14 day fasting period together with a multidisciplinary program including daily physical activity and relaxation under medical supervision. A supplementation of 200–250 kcal in the form of juices and vegetable soups is given daily [8]. The safety of this peer-reviewed fasting protocol has been documented in numerous studies [8, 26–28].

## Patients and methods

### Ethics

This prospective, observational study was approved by the medical council of Baden-Württemberg and the Ethics Committee of the Charité-University Medical Center, Berlin (application number: EA4/054/15) on 5 May 2015. The study protocol was registered on 3 June 2016 in the German Clinical Trials Register (DRKS-ID: DRKS00010111). The study took place between August 2018 and March 2019 at the BWC in Überlingen (Germany), a specialised centre for fasting therapy. The study was conducted in accordance with the principles of the Declaration of Helsinki. All participants gave their written informed consent.

### Participants

Forty volunteers (20 men and 20 women), aged 30–65 years accepted to participate in this observational study. The subjects had to undergo a fasting program of at least 14 days, including various fasting durations ranging from 13 to 26 days at the BWC. They were recruited out of the regular pool of patients. Exclusion criteria were: lipid lowering drugs, diabetes under medication, existing contraindications of fasting like anorexia nervosa, advanced kidney, liver, or cerebrovascular insufficiency, dementia and other debilitating cognitive disease, pregnancy or lactation. Moreover, subjects were not enrolled when an adequate linguistic communication was not possible in German, English or French or when the subjects participated in another clinical trial.

### Fasting program

All participants followed the fasting program of the BWC in accordance with peer-reviewed guidelines [29], supervised by nurses and specialized physicians on a daily basis. Before the beginning of the fast, subjects underwent a transition day comprising a standardized 600 kcal vegetarian mono-diet. The fasting program was conducted as previously described in detail [8]. All subjects were requested to drink 2–3 L of water or non-caloric herbal teas with an optional portion of 20 g honey every day. Moreover, they received freshly squeezed fruit juice (250 ml) at midday and a vegetable soup (250 ml) in the evening, leading to an average total daily calorie intake of 200–250 kcal and 25–35 g of carbohydrates. The breaking of fast was conducted after the third measurement on day 14. The food was slowly reintroduced on an ovo-lacto-vegetarian basis during a period of four days in which the calorie intake increased from 800 to 1600 kcal/day. The whole program was directed by certified professionals.

### Data collection

Demographic and clinical information were collected after standardized protocols of the BWC. By means of the Research Electronic Data Capture (REDCap) tool different source data were abstracted and centrally matched in a secure, web-based software platform [30]. Plausibility controls like range checks were conducted automatically during data entry.

### Clinical parameters

Body weight was measured every morning between 7:00 and 9:00 am, while subjects wore light clothing (Seca 704, Seca, Hamburg, Germany). Body height was determined with Seca 285 (Seca). At the beginning and at the end of the fast, waist circumference was measured with a measuring tape placed mid-way between the lowest rib and the iliac crest (Openmindz GmbH, Heidelberg, Germany). Blood pressure and heart rate were determined at each time point once at the non-dominant arm in the sitting position after a pause using an upper arm blood pressure monitor (Boso Carat professional, BOSCH + SOHN GmbH u. Co. KG, Jungingen, Germany).

### Categorization of the disease status at baseline

Obesity was reported when BMI was ≥ 30 kg/m^2^. Hyperlipidemia was diagnosed when LDL-C levels were ≥ 4.14 mmol/L and hypertriglyceridemia when TG levels were ≥ 1.70 mmol/L. Hypertension was diagnosed either when blood pressure (BP) values were ≥ 140/ ≥ 90 mmHg measured at the beginning of the procedure or a pre-existing disease was documented in the medical report. Type 2 diabetes was diagnosed with HbA1c levels ≥ 6.5% (> 42 mmol/mol) or according to the personal doctor’s diagnosis.

### Self-reported data

Physical activity was self-rated by the participants as a mean value indicated as hours per week before and at the end of fasting. The consumption of alcohol was documented as glasses of beer (0.3 l), wine (0.2 l) or spirits (0.02 l) per week and summed up to the amount of drinks per week.

### Safety assessments

Possible adverse events were continuously monitored and documented in an adverse event report form.

### Laboratory examinations

Blood samples were collected in the morning between 7:30 and 9:30 am at baseline, after 7 and 14 fasting days by trained medical–technical assistants. The baseline blood draw took place at the beginning of the stay at BWC either on the transition day or the first fasting day. The second blood draw was collected 7 days after the baseline blood draw. Likewise, the third sample was collected 14 days afterwards. Serum tubes (S-Monovette, 9 ml Z-Gel) were centrifuged at 3290 g (5000 rpm) for 10 min at room temperature. TC, TG, glucose, HbA1c, high-sensitivity C-reactive protein (hs-CRP) and fibrinogen were measured directly in fresh blood samples. For lipid profiles, density gradient ultracentrifugation, and NMR spectroscopy, samples were stored in aliquots at − 70 °C. All analyses have been performed within twelve months from blood collection.

### Clinical chemistry parameters

Iodixanol was supplied as 60% w/v solution (OptiPrepTM) by Axis-Shield PLC (Dundee, UK). OptiSealTM tubes (3.2 ml) were supplied by Beckman Coulter (Krefeld, Germany). Triacylglycerol, Cholesterol, LDL-Cholesterol, HDL-Cholesterol, apoB, apoA1 and lipoprotein(a) assays, calibrators and serum lipid controls were purchased from Roche (Mannheim, Germany). For lipoprotein(a), the measuring range was 7–240 nmol/L (Tina-quant^®^ Lipoprotein (a) Gen. 2, Roche). All lipid measurements were performed on the Cobas C501 from Roche. Inter- and intra-assay coefficients of variation for lipoprotein(a) are shown in Table S5. Non-HDL-C was calculated as the difference between TC and HDL-C. Glucose was measured on the Siemens ADVIA 2400. HbA1c was determined with the Tosoh G8 HPLC Analyzer. Hs-CRP was measured on the BN II Siemens nephelometric analyser. For fibrinogen, the ACL TOP (Instrumentation Laboratory) was used.

### Density gradient ultracentrifugation of lipoprotein subclasses

The procedure for the separation of lipoprotein subclasses using iodixanol originally described by Graham et al. [31, 32] was adapted as follows. 1 ml of serum, 1.24 ml Hepes buffer saline (HBS) buffer (10 mM HEPES; 0.8% NaCl; pH7.4), and 0.54 ml OptiPrepTM (60% iodixanol) were transferred into a Beckman OptiSealTM tube (3.2 ml) and thoroughly mixed by gentle, repeated over-head rotation. The mixture was carefully overlayed with distilled water to fill the tube. The tubes were housed in a Beckman TLN100 rotor and centrifuged at 400,000 g for 3 h at 15 °C with acceleration program 9 and deceleration program 9 in a Beckman Optima E-max benchtop ultracentrifuge. Gradients were collected using a Beckman gradient unloader, which pierces the tube bottom, a Watson–Marlow 520S/R precision peristaltic pump, and a Gilson (Villiers, France) fraction collector. A total of 20 fractions, each 0.16 ml, were collected by continuously pumping the gradient from the bottom of the tube to the fraction collector numbering the fractions from top (fraction 1) to bottom (fraction 20). The fractions of the gradient were pooled to obtain the following lipoprotein fractions: VLDL (fractions 1–2; iodixanol density range = 1.000–1.010 g/ml), IDL (fractions 3–4; iodixanol density range = 1.010–1.014 g/ml), LDL1 (fractions 5–8; iodixanol density range = 1.014–1.027 g/ml), LDL2 (fractions 9–11; iodixanol density range = 1.027–1.037 g/ml), LDL3 (fractions 12–13; iodixanol density range = 1.037–1.057 g/ml), HDL2 (fractions 14–17; iodixanol density range = 1.057–1.106 g/ml), and HDL3 (fractions 18–20; iodixanol density range = 1.106–1.181 g/ml). The numbering of the LDL subclasses depends on the methodology and does not match the numbering of other methods. The term "small, dense LDL" is usually used for the LDL subclass with the highest density range and the smallest diameter of the method used. In each of the lipoprotein fractions TG, TC, LDL-cholesterol, and HDL-cholesterol were measured on the Cobas c501 (Roche) with the reagent kits from Roche.

### NMR spectroscopy for analysis of lipoprotein subclasses

Lipoproteins from samples stored at − 80 °C were analyzed by NMR spectroscopy at Numares AG (Regensburg, Germany) using the AXINON LipoFIT-S100 test system according to the manufacturer's protocol. NMR spectra were recorded at a temperature of 310 K ± 0.4 K on a shielded 600 MHz Bruker Avance II + spectrometer in a fully automated process. Lipoprotein analysis was conducted via deconvolution of the broad methyl group signal at about 0.9–0.8 ppm. In this process, lipoprotein subclasses are reflected by a fixed number of predefined bell shaped (e.g. Gaussian or Lorentzian) base functions, each of which has a constant position and defined width. A least squares algorithm was used to find optimal parameters for the linear combination of base functions that minimize the deviation from the measured signal. The concentrations of lipoprotein particles and cholesterol in lipoprotein subclasses, as well as the average particle size, were calculated based on the integrals attributable to specific base functions and underwent statistical evaluation. All the measured lipid parameters are given: concentration of large VLDL particles (large VLDL-p), of all LDL particles (LDL-p), of large LDL particles (large LDL-p), of small LDL particles (small LDL-p), of all HDL particles (HDL-p), of large HDL particles (large HDL-p), of small HDL particles (small HDL-p); mean sizes of VLDL particles (VLDL-s), of LDL-p (LDL-s) and of HDL-p (HDL-s).

### Statistics

We estimated the statistical power based on the effect size for the difference in sdLDL levels observed in a previous, exploratory, pilot study with 16 individuals undergoing LF according to the same fasting protocol as presented in this study. The enrolled five men and eleven women were aged between 44 and 69 years. The pilot study was conducted within the scope of the described observational trial. In contrast to the present study, the measured fasting duration was variable and ranged between 5 and 14 days.

Effect sizes were Cohen’s *d* values calculated with the R package rstatix (version 0.6.0). They were used to calculate the statistical power with R package WebPower (version 0.5.20) in R version 4.0.0. A power of 0.92 is achieved with a sample size of 20 individuals to detect a change in sdLDL levels (Cohen’s *d* = 0.7) with a paired *t* test (alpha = 0.05).

The baseline characteristics of the patients grouped by gender were compared using the chi-squared or Fisher’s exact test for categorical variables and the *t* test for continuous variables. For the categorical variables, we reported frequencies and percentages, and the quantitative data are expressed as the mean ± standard deviation. The primary end point was set as the change in sdLDL. We applied multivariable linear mixed models for repeated measures adjusted for age and sex with unstructured covariance structure to model within-subject errors. Multiple comparison adjustment for heterogeneous variance between groups was applied. For variables that were not normally distributed, geometric means were calculated from the log-transformed variable (base e). The overall *p* value of the models (*p*), is indicated along pairwise statistical differences between the three time points as adjusted *p* values (adj-p).

We also used partial least squares discriminant analysis (PLS-DA) methods to evaluate which lipoprotein contributes the most to the effects of the fast on lipid profiles. The R package ropls version 1.20.0 was used. Prior to analysis, experimental variables were centered and unit-variance scaled. Since PLS-DA methods are prone to overfitting, we assessed the significance of our classification using permutation tests (permuted 1000 times).

All statistical analyses were performed with SAS software (version 9.4; SAS Institute Inc., Cary, North Carolina, USA). A two-sided *p* value of 0.05 was considered as statistically significant.

## Results

### Subject characteristics

**Fig. 1:**
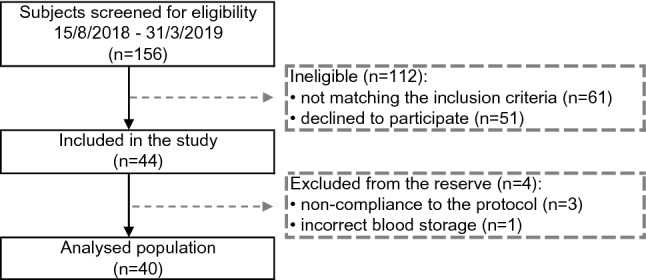
Flow chart of the selection procedure

Between August 2018 and March 2019, 156 subjects were screened for eligibility of which 44 subjects were enrolled (Fig. 1). Three individuals were excluded from analysis due to minor non-compliance and one was excluded for incorrect blood processing.

The group included 40 individuals (39 Caucasians; 50% men), aged 50.7 ± 9.5 years (range 32–65 years), with a BMI of 29.8 ± 5.5 kg/m^2^ and WC of 97.2 ± 16.0 cm; 47.5% were obese with a BMI > 30 kg/m^2^. High BP was documented in 21 individuals at baseline. Total and LDL-C were in the upper range of normal [33] (TC = 5.52 ± 1.48 mmol/L and LDL-C = 3.21 ± 1.16 mmol/L) with 16 participants (40%) classifying as hyperlipidemic. HDL-C and TG were in the normal range: 1.36 ± 0.40 mmol/L and 1.45 ± 0.6 mmol/L, respectively. Eleven participants (28%) presented hypertriglyceridemia. Overall, the baseline lipid profile did not differ between men and women. Only levels of HDL-C were significantly higher in women. Conversely, TG levels in men appeared higher but did not reach statistical significance (*p *= 0.0677). Glycemia and glycated haemoglobin (HbA1c) were in the normal range, i.e. 5.21 ± 0.66 mmol/L and 35.1 ± 5.5 nmol/mol. Only one man had a diagnosis of type 2 diabetes (Table 1).

**Table 1:** Baseline characteristics

	All	Men	Women	*p* value
*n* (%)	40	20 (50)	20 (50)	
Age, years	50.7 ± 9.5	50.6 ± 8.3	50.7 ± 10.8	0.9740•
Body mass index, kg/m^2^	29.8 ± 5.5	31.4 ± 5.4	28.3 ± 5.3	0.0798•
Weight, kg	89.6 ± 20.3	100.1 ± 18.9	79.0 ± 16.0	0.0005•
Waist circumference, cm	97.2 ± 16.0	105.5 ± 15.1	89.1 ± 12.6	0.0006•
Obesity, *n* (%)	19 (47.5)	12 (30.0)	7 (17.5)	0.1134Δ
Hypertension, *n* (%)	21 (52.5)	14 (35)	7 (17.5)	0.0267Δ
Hyperlipidemia, *n* (%)	16 (40)	8 (20.0)	8 (20.0)	1.0000Δ
Total cholesterol, mmol/L	5.52 ± 1.48	5.32 ± 0.98	5.72 ± 1.86	0.4001•
LDL-C, mmol/L	3.21 ± 1.16	3.16 ± 0.84	3.25 ± 1.43	0.8129•
HDL-C, mmol/L	1.36 ± 0.40	1.13 ± 0.24	1.58 ± 0.40	0.0002•
Hypertriglyceridemia, *n* (%)	11 (27.5)	8 (20.0)	3 (7.5)	0.1552*
Triglycerides, mmol/L	1.45 ± 0.6	1.62 ± 0.06	1.27 ± 0.59	0.0677•
Lipoprotein(a), nmol/L	10.5 [5.3; 35.4]	9.7 [5.6; 29.1]	12.3 [5.3; 62.5]	0.4470Δ
Physical activity, h/week	4.2 ± 3.5	3.6 ± 2.4	4.7 ± 2.7	0.3210•
Type 2 diabetes, *n* (%)	1 (2.5)	1 (2.5)	0 (0.0)	1*
Glucose, mmol/L	5.21 ± 0.66	5.37 ± 0.67	5.05 ± 0.61	0.1117•
HbA1c, mmol/mol	35.1 ± 5.5	36.3 ± 6.9	33.9 ± 3.5	0.1767•
Drinks, glasses/week	9.2 ± 3.9	13.4 ± 21.2	5.0 ± 5.1	0.1092•
Physical activity, h/week	4.2 ± 3.5	3.6 ± 2.4	4.7 ± 2.7	0.3210•
Ethinicity (% Caucasians)	39 (97.5)	19 (95.0)	20 (100.0)	1*

The baseline characteristics of the patients grouped by gender were compared using the chi-squared or Fisher exact test for categorical variables and the *t* test for continuous variables. Criteria of baseline disease status: Obesity (BMI ≥ 30 kg/m^2^); hypertension (blood pressure ≥ 140/ ≥ 90 mmHg or a pre-existing diagnosis); hypercholesterolemia (LDL-C ≥ 4.14 mmol/L); hypertriglyceridemia (TG ≥ 1.70 mmol/L); diabetes type 2 (HbA1c levels ≥ 42 mmol/mol or a pre-existing diagnosis).Values in mmol/L can be converted to mg/dL by multiplication with 38.6 for cholesterol and 88.5 for TG [64]. Continuous variables are expressed as mean ± standard deviation (SD), as median [first quartile-third quartile] if not normally distributed; or as frequency and percentage for categorical variables

*p* value from *Fisher exact test

^•^*t* test

^Δ^Chi-squared test

### Impact of fasting on anthropometric measurements, lipid and non-lipid cardiovascular risk biomarkers

Fasting resulted in a significant weight loss of 4.5 ± 0.2 kg after 7 days and 7.0 ± 0.3 kg after 14 days (*p *< 0.0001) (Table 2 and S1 for gender differences). Waist circumference showed a mean reduction of 7.5 ± 0.4 cm at the end of fasting (*p *< 0.0001). Total cholesterolemia showed a slight, non-significant decrease after the first 7 days of fasting (5.32 ± 0.25 mmol/L; adj-p = 0.2459) and the decrease became significant after 14 days (from 5.52 ± 0.23 to 4.57 ± 0.27 mmol/L; adj-p < 0.0001; Table 2). Levels of plasma TGs were also reduced during the intervention, decreasing after 7 days from 1.45 ± 0.1 mmol/L to 1.15 ± 0.05 mmol/L (adj-p = 0.0065) and after 14 days to 1.1 ± 0.04 mmol/L (adj-*p *= 0.0025). Men had higher TG values (*p *= 0.0466). Levels of LDL-C and non-HDL-C showed a slight, non-significant decrease after 7 days (respectively, *p *= 0.9890 and *p *= 0.8494); this became significant after 14 days (LDL-C: adj-p < 0.0001; non-HDL-C: adj-*p *= 0.0002). Lipoprotein(a) levels increased after 7 days and subsequently decreased (*p *< 0.0001). Levels of ApoAI, higher in women at baseline (*p *= 0.0006), decreased significantly during fasting (*p *= 0.0431). ApoB levels showed a slight, non-significant increase after 7 days of fasting (adj-p = 0.0808), returning to basal after 14 days (adj-*p *= 0.9807). Fibrinogen and hs-CRP (Table 2) were in the normal range at baseline. A tendency to increase was observed in both parameters after 7 days, followed by a significant decrease after 14 days (Fibrinogen: *p *= 0.0056; hs-CRP: *p *= 0.0029) when values returned to baseline. Physical activity, recommended by the program, increased during fasting from 4.2 ± 0.6 h/week at baseline to 13.9 ± 1.1 h/week at the end of trial (*p *< 0.0001).

**Table 2:** Changes in lipids, lipoprotein subclasses and ASCVD risk biomarkers during fasting

Parameter	All			
	0 d	7 d	14 d	*p* value
Weight, kg	89.6 ± 3.2^a,b^	85.0 ± 3.1^c^	82.5 ± 3.0	< 0.0001
BMI, kg/m^2^	29.8 ± 0.9^a,b^	28.3 ± 0.8^c^	27.5 ± 0.8	< 0.0001
Waist circumference, cm	97.3 ± 2.5	–	89.8 ± 2.4	< 0.0001
Physical exercise, h/week	4.2 ± 0.6	–	13.9 ± 1.1	< 0.0001
TC, mmol/L	5.52 ± 0.23^b^	5.32 ± 0.25^c^	4.57 ± 0.27	< 0.0001
TG, mmol/L	1.45 ± 0.1^a,b^	1.15 ± 0.05	1.1 ± 0.04	0.0041
VLDL-C, mmol/L	0.42 ± 0.03^a,b^	0.27 ± 0.02	0.25 ± 0.02	< 0.0001
VLDL-TG, mmol/L	0.7 ± 0.01^a,b^	0.34 ± 0.01	0.35 ± 0.01	< 0.0001
IDL-C, mmol/L	0.18 ± 0.01^b^	0.16 ± 0.0	0.15 ± 0.01	0.0135
IDL-TG, mmol/L	0.07 ± 0.01	0.07 ± 0.01	0.07 ± 0.01	0.5778
LDL-C, mmol/L	3.21 ± 0.18^b^	3.19 ± 0.22^c^	2.48 ± 0.21	< 0.0001
LDL1-C, mmol/L	1.00 ± 0.07^b^	1.06 ± 0.08^c^	0.84 ± 0.08	< 0.0001
LDL2-C, mmol/L	1.31 ± 0.08^b^	1.30 ± 0.09^c^	1.02 ± 0.09	< 0.0001
LDL3-C, mmol/L	0.89 ± 0.06^b^	0.83 ± 0.06^c^	0.63 ± 0.05	< 0.0001
LDL-TG, mmol/L	0.32 ± 0.01^a,b^	0.46 ± 0.02	0.44 ± 0.02	< 0.0001
LDL1-TG, mmol/L	0.14 ± 0.01^a,b^	0.21 ± 0.01	0.2 ± 0.01	< 0.0001
LDL2-TG, mmol/L	0.09 ± 0^a,b^	0.14 ± 0.01	0.13 ± 0.01	< 0.0001
LDL3-TG, mmol/L	0.08 ± 0^a,b^	0.11 ± 0	0.1 ± 0	< 0.0001
HDL-C, mmol/L	1.36 ± 0.06^a,b^	1.22 ± 0.06	1.19 ± 0.06	0.0014
HDL2-C, mmol/L	1.10 ± 0.06^a,b^	1.01 ± 0.05	1.00 ± 0.06	0.0261
HDL3-C, mmol/L	0.25 ± 0.01^a,b^	0.21 ± 0.01	0.19 ± 0.01	< 0.0001
HDL-TG, mmol/L	0.2 ± 0.01	0.21 ± 0.01	0.2 ± 0.01	0.4612
HDL2-TG, mmol/L	0.13 ± 0	0.13 ± 0	0.13 ± 0.01	0.2659
HDL3-TG, mmol/L	0.07 ± 0.01	0.07 ± 0.01	0.07 ± 0.01	0.3705
non-HDL-C, mmol/L	4.17 ± 0.22^b^	4.10 ± 0.24^c^	3.38 ± 0.24	< 0.0001
Lipoprotein(a), nmol/L^d^	11.7 ± 1.3^a^	21.7 ± 1.3^c^	14.0 ± 1.3	< 0.0001
ApoA1, µmol/L	46.4 ± 3.6 ^b^	42.8 ± 3.6	39.3 ± 3.6	0.0431
ApoB, µmol/L	1.6 ± 0.2	2 ± 0.2 ^c^	1.6 ± 0.2	0.0076
Fibrinogen, mg/dL	337.5 ± 18.0	363.9 ± 17.0^c^	328.9 ± 15.2	0.0056
hs-CRP, nmol/L	17.1 ± 12.4	24.8 ± 11.4^c^	18.1 ± 11.4	0.0029

The overall p value is calculated by means of linear mixed models for repeated measures, with unstructured covariance structure to model within-subjects errors. Multiple comparison adjustment for heterogeneous variance between group was applied. Values are indicated as mean ± SEM. Values in mmol/L can be converted to mg/dL by multiplication with 38.6 for cholesterol and 88.5 for TG [64].

Statistically significant differences between the three time points by means of adjusted *p* values are indicated with

^a^0 d (baseline) vs. 7 d

^b^0 d vs. 14 d

^c^7 d vs. 14 d

^d^Reported as geometric means ± SEM

### Lipoprotein subclasses analysis by density gradient ultracentrifugation

Since circulating lipoprotein particles vary in size, density, and lipid composition, each showing a different atherogenic pattern, we analysed the impact of long-term fasting on lipoprotein subclasses along with their cholesterol and TG contents (Table 2 and S2 for gender differences).

#### VLDL

A VLDL-C decrement of − 0.15 ± 0.03 mmol/L was reached 7 days after food cessation, an effect stable after 14 days of fasting (− 0.16 ± 0.03 mmol/L; *p *< 0.0001). The TG content of VLDL was reduced by − 0.44 ± 0.07 mmol/L and − 0.46 ± 0.08 mmol/L after 7 and 14 days (*p *< 0.0001), respectively.

#### IDL

IDL-C, the catabolic product of VLDL-C fell after 7 and 14 days of fasting by − 0.01 ± 0.01 mmol/L and − 0.03 ± 0.01 mmol/L (*p *= 0.0135), respectively. Only a slight, non-significant increase in IDL-TG content was found (*p *= 0.5778) but levels in men appeared significantly higher overall (*p *= 0.0409).

#### LDL

Two weeks of fasting led to a drop in the cholesterol content of LDL1, LDL2 and LDL3 by − 0.16 ± 0.05 mmol/L, − 0.30 ± 0.06 mmol/L, and − 0.27 ± 0.05 mmol/L, respectively (each *p *< 0.0001; Table 2). These changes were accompanied by significant rises in the TG content by 0.06 ± 0.01 mmol/L (LDL1), 0.04 ± 0.01 mmol/L (LDL2) and 0.02 ± 0.01 mmol/L (LDL3; each *p *< 0.0001).

#### HDL

In the whole group, HDL2-C decreased after one week by − 0.10 ± 0.04 mmol/L and remained stable up to 14 days (− 0.11 ± 0.04 mmol/L; *p *= 0.0261), an effect apparently driven by the higher baseline levels of women (*p *= 0.0006). A similar trend was found in the analysis of HDL3-C, decreasing by − 0.04 ± 0.01 mmol/L (7 days) and − 0.06 ± 0.01 mmol/L (14 days; *p *< 0.0001), again women had higher baseline values (*p *= 0.0065). No differences were found in the HDL-TG content (HDL2-TG: *p *= 0.2659; HDL3-TG: *p *= 0.3705).

Ratios that reflect the lipid content within the lipoproteins can be found in the supplementary material (Table S3).

Altogether, these results suggest that reductions in plasma cholesterol levels are driven by a reduction in cholesterol in all lipoprotein subclasses but predominantly in LDL-C. Conversely, reductions in plasma triglyceride levels are exclusively explained by reductions in VLDL-TG levels.

### NMR lipoprotein particle size and concentrations

In addition to the ultracentrifugation, a NMR analysis was conducted to get further insights on particle size and concentrations (Table 3 and S4 for gender differences). Fasting did not affect mean particle sizes of LDL (LDL-s; *p* = 0.1657) and VLDL (VLDL-s; *p* = 0.5181). Instead, HDL size (HDL-s; *p* < 0.0001) increased, particularly in men (*p* = 0.005).

**Table 3:** Changes in lipoprotein size and particle concentration during fasting

Parameter	All			
	0 d	7 d	14 d	*p* value
VLDL-s, nm	47.57 ± 0.69	47.37 ± 0.34	47.63 ± 0.33	0.5181
LDL-s, nm	21.10 ± 0.07	21.11 ± 0.05	21.07 ± 0.05	0.1657
HDL-s, nm	9.07 ± 0.09^a,b^	9.24 ± 0.08^c^	9.39 ± 0.07	< 0.0001
Large VLDL-p, nmol/L	4.74 ± 1.15^a,b^	1.94 ± 1.08	1.90 ± 1.04	< 0.0001
LDL-p, nmol/L	1479.3 ± 73.9^b^	1477.7 ± 83.5^c^	1186.2 ± 76.8	< 0.0001
Large LDL-p, nmol/L	889.3 ± 53.1^b^	823.0 ± 57.4^c^	630.4 ± 54.2	< 0.0001
Small LDL-p, nmol/L	590.0 ± 48.3	654.8 ± 42.0^c^	555.2 ± 34.8	0.0001
HDL-p, nmol/L	36,241.0 ± 1156.5^a,b^	30,920.9 ± 663.2^c^	28,312.8 ± 613.1	< 0.0001
Large HDL-p, nmol/L	6703.4 ± 699.1	6929.1 ± 666.9	7507.7 ± 561.8	0.0419
Small HDL-p, nmol/L	29,537.6 ± 1123.6^a,b^	23,991.8 ± 758.1^c^	20,792.8 ± 563.7	< 0.0001

The overall *p* value is calculated by means of linear mixed models for repeated measures, with unstructured covariance structure to model within-subjects errors. Multiple comparison adjustment for heterogeneous variance between group was applied. Values are indicated as mean ± SEM.

Statistically significant differences between the three time points by means of adjusted *p* values are indicated with

^a^0 d (baseline) vs. 7 d

^b^0 d vs. 14 d

^c^7 d vs. 14 d.

The concentrations in large VLDL-p decreased significantly (− 4.10 ± 1.22 nmol/L) after 7 fasting days and by − 5.18 ± 1.26 nmol/L after 14 days (*p* < 0.0001). Total LDL-p showed a slight, non-significant decrease after the first week of fasting (adj-p = 0.9990) but decreased significantly after 14 days (adj-p < 0.0001), with a drop in both large (− 244.13 ± 39.45 nmol/L; *p* < 0.0001) and small LDL-p (-38.45 ± 44.04 nmol/L; *p* < 0.0001). In particular, concentrations in small LDL-p differed between genders with a more pronounced decrement in men (*p* = 0.0047). Although the concentrations in total HDL-p decreased significantly (*p* < 0.0001), during fasting regardless of gender (*p* = 0.2193), large HDL-p increased after 7 days and after 14 days (p = 0.0419). Conversely, there was a decrease in small HDL-p during fasting in the whole population (− 8700.6 ± 867.6 nmol/L; *p* < 0.0001).

**Fig. 2:**
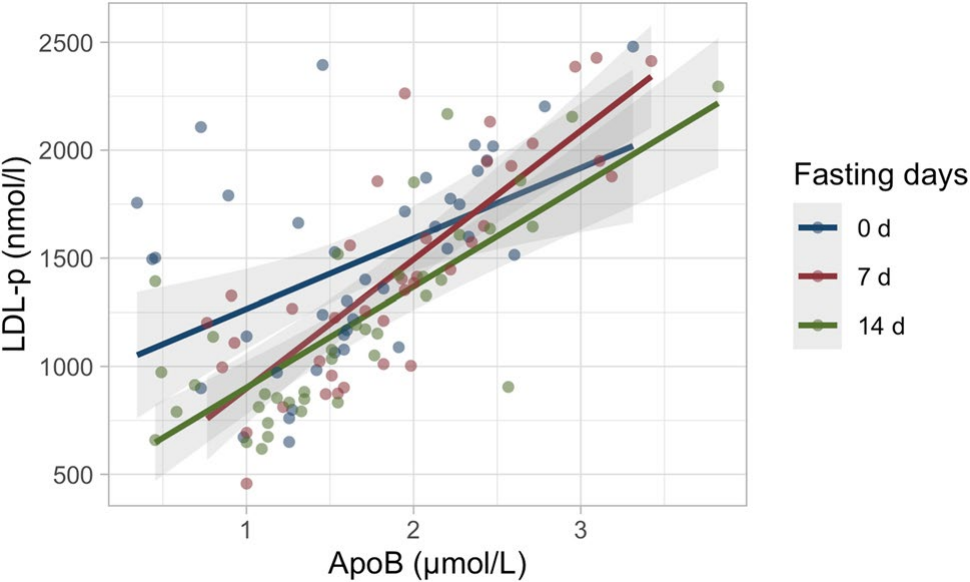
Changes in apoB were associated with changes in LDL-p concentrations at baseline, after 7 and 14 fasting days. Dot plots show the correlation for the parameters between the different time points (baseline, red; 7 fasting days, green; 14 fasting days, blue)

Furthermore, it was evaluated whether changes in LDL-p concentrations were associated with changes in apoB. Figure 2 shows a high correlation between increased LDL-p concentrations and increased apoB (*p* < 0.0001).

### Relative contribution of the different lipoproteins to the lipoprotein changes observed during fasting

To dissect out the relative contribution of each lipoprotein in the differences observed during fasting, a partial least squares discriminant analysis (PLS-DA) was performed. Lipoprotein profiles at baseline or during fasting were well separated with no outliers (Fig. S1). Permutation testing showed that the difference between the two was statistically significant (pR2Y < 0.001 and pQ2 < 0.001), lipoproteins contributing most to this difference (VIP scores > 1.5) were VLDL, LDL2 and LDL1. Although the separation between different classes by NMR was statistically significant, no VIP score exceeded 1.5. Overall, this multivariate analysis suggests that the largest lipoproteins (VLDL, LDL1 and 2) were the most affected by fasting, HDL lipoproteins being the least affected.

## Discussion

Nutritional strategies, such as IF, alternate-day fasting and LF (up to three weeks), have been shown to affect the CV risk markers as previously reviewed by our group [1]. Since blood lipids play a causal role in the etiology of coronary heart disease, detailed lipid-lipoprotein determinations can be used to assess risk changes in a non-invasive way. By contrast, other non-invasive vascular evaluations, such as the carotid intima media thickness, are unlikely to be affected by short or medium-term interventions [34]. A major novelty of this report is the reduction of apoB-containing lipoproteins following LF up to 14 days, with LDL3-C and VLDL-TG being the most affected and HDL the least modified.

Fasting is defined by a cessation of food intake, thus interrupting exogenous lipid intake. The transport of dietary lipids from the intestine to peripheral tissues and liver is thus presumably halted. Subsequently, neither chylomicrons nor chylomicron remnants are formed. At the same time, free fatty acids from the adipose tissue are mobilized [35]. Thus, the synthesis of VLDL may be reduced. A rapid decline in circulating VLDL-C, concomitant with a decrease in IDL-C was documented in our cohort. Not all LDL-C subclasses were reduced after 7 days of fasting, whereas significant decreases were documented after 14 fasting days. This suggests that longer periods of fasting will possibly enhance the benefits.

**Fig. 3:**
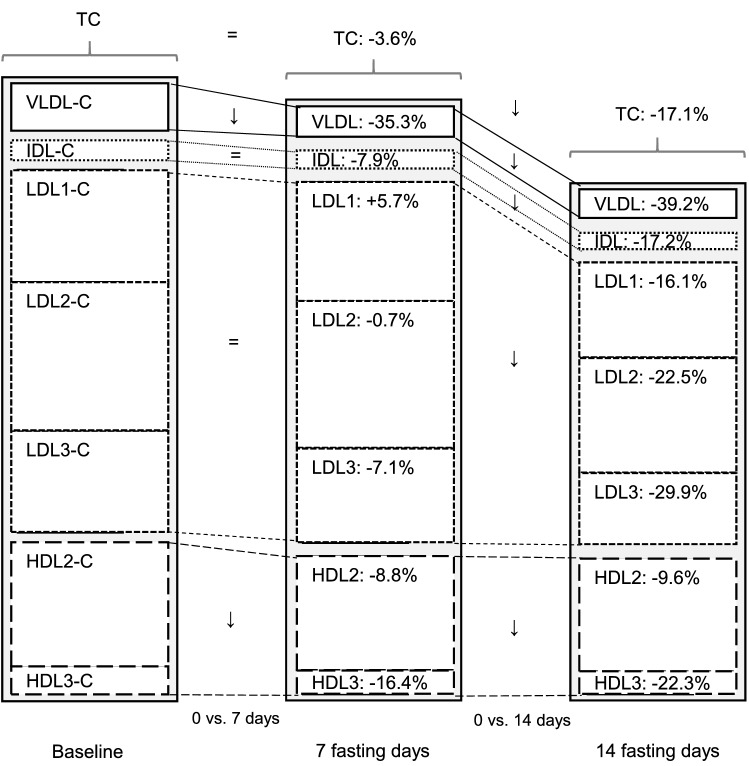
Changes of cholesterol concentration in lipoprotein subclasses before and after 7 and 14 days of fasting. Significant changes between baseline and 7 days as well as baseline and 14 days of fasting are indicated with an arrow. Non-significant changes are indicated with an equal sign

TC and LDL-C have been traditionally considered as single ASCVD lipid risk factors, but nowadays the role of LDL sub-fractions has gained interest [20]. SdLDLs have a prolonged residence time in the circulation with extensive inter-particle transfer via the cholesteryl ester transfer protein (CETP), increasing risk of plaque formation. Furthermore, the low affinity of sdLDL for the LDL receptors in the liver is also leading to a lower reuptake and longer residence in the circulation [22]. In the present study, we showed an early percent reduction of sdLDL after 7 days of fasting and still more after 14 days. SdLDL particles confer a greater atherogenic risk than larger, less dense LDL particles [36], being more avidly taken up by macrophages than larger, less dense LDL, being more susceptible to oxidative modification, having a greater propensity for transport into the arterial subendothelial space, and for binding potential to arterial wall proteoglycans [21]. The exposure of LDL-p to oxidative stress in endothelial cells involves the action of reactive oxygen species [37] with recruitment of several cell types, including endothelial cells, monocyte-macrophages (leading to foam cell formation), T lymphocytes, and fibroblasts [38]. In a previous study of a ten-day fast with the same program [39], LF reduced lipid peroxidation and concomitantly raised the antioxidant capacity as well as redox biomarkers, thus antagonizing LDL oxidation [39, 40]. A higher LDL-p concentration has been associated with cardiovascular disease (CVD) incidence, but since studies have not determined whether any measures of LDL sub-fractions add incremental benefit to the traditional risk factor assessment [41], the aim of the present study was to determine LDL sub-fraction changes after the LF dietary intervention and their possible impact beyond that of the traditional risk factors. As suggested by other authors, apoB-containing particles including lipoprotein(a) appear to provide a better prediction of risk versus LDL-C, since many individuals with atherogenic dyslipidemia have increased concentrations of LDL-p without a rise in LDL-C [42]. Aside from sdLDL, it is assumed that all LDL-p are atherogenic [43]. This is shown in the case of patients with familial hypercholesterolemia displaying a predominance of large, buoyant LDL particles and early atherosclerosis [44]. All measured LDL subclasses are reduced by 20% or more at the end of 14 days of fasting (Fig. 3). Since LDL1-C is less atherogenic than LDL3-C [45], LF appears to be linked to a reduced lipoprotein-associated risk profile.

Each circulating atherogenic lipoprotein particle contains one apoB molecule, but the amount of cholesterol (especially in LDL-p) and the amount of TG (especially in VLDL-p) can vary extensively among particles. Whilst the concentration of LDL-C and TG quantifies their concentrations in circulating lipoproteins, they do not precisely quantify the concentration of atherogenic particles, being instead theoretically directly proportional to the concentrations of apoB molecules [46, 47]. In the present study, we observed a discordance between apoB and LDL-p, as earlier described by Varvel et al. [48]. This could be due to the efficiency of current apoB immunoassays that may vary with respect to particle size or shape because of conformational changes in the binding epitope of apoB as the particle shrinks or distorts [48].

Some epidemiological studies have reported that the levels of non-HDL-C, which includes cholesterol of TG-rich lipoproteins, could be a better risk predictor in individuals with hypertriglyceridemia [49]. Non-HDL-C concentrations have been strongly associated with long-term risk of atherosclerotic cardiovascular disease [50]. Most adults with elevated non–HDL-C early in life continue to have high non–HDL-C over the entire life course, leading to significantly increased CVD risk [51]. The results of the present study indicative of a large decrement in non-HDL-C are in line with strategies to improve CV risk [50].

Lipoprotein-associated CV risk, besides being dependent on LDL-C, is determined by a cluster of metabolic abnormalities, referred to as the “atherogenic lipid phenotype”, characterized by elevated fasting and post-prandial TG-rich lipoprotein levels and their remnants, increased sdLDL and decreased HDL [15, 52]. The major TG-containing lipoproteins in fasted subjects are VLDL (*d* < 1.006) and, to a lesser extent, IDL (*d* > 1.006 but < 1.019), both containing apoB-100. Since the slowly metabolized sdLDL are derived from the lipolysis of the large VLDL species [15], the observed reduction of VLDL is clinically important. VLDL remnants are direct substrate for atherosclerotic plaque development, rather than TG [53]. In patients with CVD, elevated levels of VLDL-C account for one-half of the risk of myocardial infarction [54]. Moreover, the transient rise we found in lipoprotein(a) seems in line with previous evidence reporting that reduction of saturated fat was associated with an increase in lipoprotein(a) levels, despite a decrement in LDL-C [55].

The protective effects of HDL are well known but the role of two major HDL sub-fractions (HDL2-C, HDL3-C) is unclear. Some studies suggest that HDL2-C is more atheroprotective, while HDL3-C is less atheroprotective or neutral on CV risk [56, 57], whereas other studies suggest that HDL3-C is more atheroprotective [58]. There is indication that HDL2 is atheroprotective because of a higher vasodilatory activity [59], whereas HDL3 is responsible for the major activity of HDL, *i.e.,* tissue cholesterol removal [60]. There is also evidence that both sub-fractions correlate with reduced risk of CV events [61]. Our data showed a reduction in both HDL2 and HDL3 levels. By NMR analysis, small HDL (mainly identifiable with HDL3) showed a larger reduction whereas the larger HDL particles were significantly raised. This is in line with the observed drop in apoA1 reflecting a decrease in the precursor of HDL [62]. The small reduction of total HDL-C can be well ascribed to the apparently raised CETP activity, resulting in an increased transfer-exchange of TG for cholesterol between VLDL and HDL [63].

Several methodologies for the assessment of lipoprotein composition exist, limiting the comparability of different study results. As reported in the consensus-based recommendations from European Atherosclerosis Society (EAS) and European Federation of Clinical Chemistry and Laboratory Medicine (EFLM), there is a need to standardize and validate advanced lipoprotein tests, such as NMR- or ion mobility-based LDL- and VLDL-particle numbers and size [64]. Overall, the application of NMR measurements combined with the ultracentrifugation (*i.e.,* the traditional method) showed complementary results in the present study. The use of high-throughput testing with NMR should be taken into consideration for future clinical laboratory testing, because ultracentrifugation is restricted by the need of manual sample preparation, hindering the routine service.

A limitation of the present study is that patients were from Caucasian ethnicity with one exception. Furthermore, we did not measure CETP activity and can thus only hypothesize that this was reduced. Considering that CETP facilitates the net movement of cholesteryl ester from HDL to VLDL and LDL in exchange for TG [65], we found that fasting reduced the cholesterol content and increased that of TG in LDL. In line with this hypothesis, a rise in the velocity of CE transfer from HDL to VLDL and LDL has been described post-prandially after fat intake [66]. Moreover, the lipoprotein lipase activity was not, measured: the insulin drop during fasting [27, 67] could reduce the insulin-dependent activity of the enzyme. When TG levels at baseline are ≥ 1.70 mmol/L, the decrease in TG levels is much more pronounced than with lower levels. Future studies should investigate individuals with and without hypertriglyceridemia. Feasibility and safety of this fasting program were documented in a large cohort [8], the follow-up, monitoring long-term lipoprotein changes can provide further important information. Finally, the coefficient of variation greater than 30% for lipoprotein(a) levels below 20 nmol/L could have affected the results found at 7 days. This could be a consequence of weight loss [68].

In conclusion, exposure to LF was followed by favourable changes in most plasma lipids, lipoprotein levels and lipoprotein subclasses (Fig. 3). In particular, the reduction of TG and of sdLDL appears to clearly indicate the potential CV benefit of this fasting intervention, to be confirmed in future follow-up studies.

## Supplementary Information

Below is the link to the electronic supplementary material.

Supplementary file1 (DOCX 3475 KB)

## Data Availability

The dataset analyzed during the current study is available from the corresponding author on reasonable request.
