# Supplementary material for: Long-term fasting improves lipoprotein-associated atherogenic risk in humans
Source: Eur J Nutr. 2021 May 7;60(7):4031–44. doi: 10.1007/s00394-021-02578-0 (PMC8437871; doi:10.1007/s00394-021-02578-0)
Supplement: Supplementary file 1 — Supplementary file1 (DOCX 3475 KB) [file 394_2021_2578_MOESM1_ESM.docx]

**Electronic Supplementary Material**

***European Journal of Nutrition***

**Long-term fasting improves lipoprotein-associated atherogenic risk in humans**

Franziska Grundler^1,2^, Dietmar Plonné^3^, Robin Mesnage^4^, Diethard Müller^5^, Cesare R Sirtori^6^, Massimiliano Ruscica^6^, Françoise Wilhelmi de Toledo^1^

^1^ Buchinger Wilhelmi Clinic, Wilhelm-Beck-Straße 27, 88662 Überlingen, Germany

^2^ Charité-Universitätsmedizin Berlin, corporate member of Freie Universität Berlin, Humboldt-Universität zu Berlin, and Berlin Institut of Health, Berlin, Germany

^3^ MVZ Humangenetik Ulm, Karlstraße 31-33, 89073 Ulm, Germany

^4^ Gene Expression and Therapy Group, King’s College London, Faculty of Life Sciences & Medicine, Department of Medical and Molecular Genetics, 8th Floor, Tower Wing, Guy’s Hospital, Great Maze Pond, London, SE1 9RT, United Kingdom

^5^ MVZ Labor Ravensburg, Elisabethenstraße 11, 88212 Ravensburg, Germany

^6^ Department of Pharmacological and Biomolecular Sciences, Università degli Studi di Milano, Milan, Italy

Corresponding author: Françoise Wilhelmi de Toledo

Email: [francoise.wilhelmi@buchinger-wilhelmi.com](mailto:francoise.wilhelmi@buchinger-wilhelmi.com)

**Table S1. Changes in lipids and CV risk biomarkers for men and women during fasting.** Statistically significant differences between the three time points by means of adjusted p-values are indicated with ^a^: 0 d (baseline) vs. 7 d; ^b^: 0 d vs. 14 d and ^c^: 7 d vs. 14 d. The overall p-value is calculated by means of linear mixed models for repeated measures, with unstructured covariance structure to model within-subjects errors. Multiple comparison adjustment for heterogeneous variance between group was applied. Values are indicated as mean±SEM.

|  | **all** |  |  |  | **men** |  |  | **women** |  |  | **overall p-value** |  |  |
| --- | --- | --- | --- | --- | --- | --- | --- | --- | --- | --- | --- | --- | --- |
| **Parameter** | **0 d** | **7 d** | **14 d** | **p-value** | **0 d** | **7 d** | **14 d** | **0 d** | **7 d** | **14 d** | **time** | **sex** | **time* sex** |
| Weight, kg | 89.6±3.2^a,b^ | 85.0±3.1^c^ | 82.5±3.0 | <0.0001 | 100.1±3.9^a,b^ | 95.0±3.8^c^ | 92.2±3.7 | 79.0±3.9^a,b^ | 75.1±3.8^c^ | 72.9±3.7 | <0.0001 | 0.0005 | 0.0038 |
| BMI, kg/m^2^ | 29.8±0.9^a,b^ | 28.3±0.8^c^ | 27.5±0.8 | <0.0001 | 31.4±1.2^a,b^ | 29.7±1.2^c^ | 28.9±1.1 | 28.3±1.2^a,b^ | 26.9±1.2^c^ | 26.1±1.1 | <0.0001 | 0.0877 | 0.2084 |
| Waist circum-ference, cm | 97.3±2.5 | - | 89.8±2.4 | <0.0001 | 105.5±3.1 | - | 97.5±2.9 | 89.1±3.1 | - | 82.1±2.9 | <0.0001 | 0.0005 | 0.2923 |
| Physical exercise, h/week | 4.2±0.6 | - | 13.9±1.1 | <0.0001 | 3.6±0.8 | - | 14.9±1.5 | 4.7±0.8 | - | 13.0±1.5 | <0.0001 | 0.7874 | 0.1944 |
| TC, mmol/L | 5.52±0.23^b^ | 5.32±0.25^c^ | 4.57±0.27 | <0.0001 | 5.32±0.33^b^ | 5.19±0.36^c^ | 4.42±0.38 | 5.72±0.33^b^ | 5.45±0.25^c^ | 4.73±0.38 | <0.0001 | 0.5045 | 0.8179 |
| TG, mmol/L | 1.45±0.1^a,b^ | 1.15±0.05 | 1.1±0.04 | 0.0041 | 1.62±0.13^a,b^ | 1.17±0.07 | 1.17±0.06 | 1.27±0.13 | 1.12±0.07 | 1.03±0.06 | 0.0041 | 0.0466 | 0.1968 |
| VLDL-C,  mmol/L | 0.42±0.03^a,b^ | 0.27±0.02 | 0.25±0.02 | <0.0001 | 0.45±0.05^a,b^ | 0.30±0.03 | 0.28±0.03 | 0.39±0.05^a,b^ | 0.25±0.03 | 0.23±0.03 | <0.0001 | 0.1770 | 0.9676 |
| VLDL-TG,  mmol/L | 0.7±0.01^a,b^ | 0.34±0.01 | 0.35±0.01 | <0.0001 | 0.83±0.01^a,b^ | 0.37±0.01 | 0.39±0.01 | 0.59±0.01^a,b^ | 0.31±0.01 | 0.32±0.01 | <0.0001 | 0.1248 | 0.6978 |
| IDL-C,  mmol/L | 0.18±0.01^b^ | 0.16±0.01 | 0.15±0.01 | 0.0135 | 0.18±0.02 | 0.17±0.02 | 0.16±0.02 | 0.18±0.02 | 0.16±0.02 | 0.14±0.02 | 0.0146 | 0.5867 | 0.7225 |
| IDL-TG,  mmol/L | 0.07±0.01 | 0.07±0.01 | 0.07±0.01 | 0.5778 | 0.09±0.01 | 0.08±0.01 | 0.08±0.01 | 0.08±0.01 | 0.08±0.01 | 0.08±0.01 | 0.9924 | 0.0409 | 0.2216 |
| LDL-C,  mmol/L | 3.21±0.18^b^ | 3.19±0.22^c^ | 2.48±0.21 | <0.0001 | 3.16±0.26^b^ | 3.22±0.31^c^ | 2.39±0.30 | 3.25±0.26^b^ | 3.16±0.31^c^ | 2.58±0.30 | <0.0001 | 0.8520 | 0.1732 |
| LDL-TG,  mmol/L | 0.32±0.01^a,b^ | 0.46±0.02 | 0.44±0.02 | <0.0001 | 0.34±0.02^a,b^ | 0.46±0.03 | 0.46±0.03 | 0.3±0.02^a,b^ | 0.45±0.03 | 0.42±0.03 | <0.0001 | 0.3335 | 0.6539 |
| HDL-C,  mmol/L | 1.36±0.06^a,b^ | 1.22±0.06 | 1.19±0.06 | 0.0014 | 1.13±0.08 | 1.02±0.07 | 1.05±0.08 | 1.58±0.08^a,b^ | 1.41±0.07 | 1.34±0.08 | 0.0011 | 0.0004 | 0.0724 |
| HDL-TG,  mmol/L | 0.2±0.01 | 0.21±0.01 | 0.2±0.01 | 0.4612 | 0.22±0.01 | 0.21±0.01 | 0.2±0.02 | 0.19±0.01 | 0.21±0.01 | 0.19±0.02 | 0.4653 | 0.3161 | 0.4003 |
| LDL-C/HDL-C | 2.5±0.2^b^ | 2.8±0.2^c^ | 2.2±0.2 | <0.0001 | 2.9±0.2 | 3.2±0.3 | 2.4±0.3 | 2.1±0.2 | 2.4±0.3^c^ | 2.0±0.3 | <0.0001 | 0.0568 | 0.0011 |
| LDL-TG/  HDL-C | 0.1±0.0^a,b^ | 0.1±0.0^c^ | 0.2±0.0 | <0.0001 | 0.1±0.0^a,b^ | 0.1±0.0^c^ | 0.2±0.0 | 0.1±0.0^a,b^ | 0.1±0.0 | 0.2±0.0 | <0.0001 | 0.3941 | 0.1761 |
| LDL-TG/  HDL-TG | 1.6±0.1^a,b^ | 2.2±0.1 | 2.4±0.1 | <0.0001 | 1.6±0.1^a,b^ | 2.3±0.1 | 2.4±0.2 | 1.6±0.1^a,b^ | 2.2±0.1 | 2.3±0.2 | <0.0001 | 0.6592 | 0.8109 |
| HDL-TG/  HDL-C | 0.2±0.5^a^ | 0.2±0.5 | 0.2±0.5 | 0.0384 | 0.2±0.5 | 0.2±0.5 | 0.2±0.5 | 0.1±0.5 | 0.2±0.5 | 0.2±0.5 | 0.0394 | 0.0004 | 0.5352 |
| VLDL-C/VLDL-TG | 0.5±2.5^a,b^ | 0.7±2.5 | 0.7±2.5 | <0.0001 | 0.5±2.5^a^ | 0.7±2.5 | 0.7±2.5 | 0.7±2.5 | 0.7±2.5 | 0.7±2.5 | 0.0001 | 0.7716 | 0.0674 |
| TG/HDL-C | 1.2±0.1 | 1±0 | 1±0 | 0.2 | 1.5±0.1 | 1.2±0.1 | 1.2±0.1 | 0.9±0.1 | 0.9±0.1 | 0.8±0.1 | 0.1945 | 0.0001 | 0.2570 |
| TC/HDL-C | 4.0±0.2 | 4.2±0.2^c^ | 3.7±0.2 | <0.0001 | 4.5±0.2 | 4.7±0.3^c^ | 4.0±0.3 | 3.4±0.2 | 3.7±0.3^c^ | 3.4±0.3 | <0.0001 | 0.0206 | 0.0340 |
| non-HDL-C, mmol/L | 4.17±0.22^b^ | 4.10±0.24^c^ | 3.38±0.24 | <0.0001 | 4.19±0.32^b^ | 4.17±0.34^c^ | 3.38±0.34 | 4.14±0.32^b^ | 4.04±0.34^c^ | 3.39±0.34 | <0.0001 | 0.9031 | 0.6800 |
| Lipoprotein(a),  nmol/L* | 11.7±1.3^a^ | 21.7±1.3^c^ | 14.0±1.3 | <0.0001 | 8.9±1.5^a^ | 17.8±1.4^c^ | 12.5±1.4 | 15.5±1.5^a^ | 26.7±1.4^c^ | 15.6±1.4 | <0.0001 | 0.4089 | 0.3170 |
| ApoA1,  µmol/L | 46.4±3.6 | 42.8±3.6 | 39.3±3.6 | 0.0431 | 42.8±3.6 | 35.7±3.6 | 35.7±3.6 | 50±3.6 | 53.6±3.6 | 42.8±3.6 | 0.0389 | 0.0006 | 0.2397 |
| ApoB,  µmol/L | 1.6±0.2 | 2±0.2 | 1.6±0.2 | 0.0076 | 1.8±0.2 | 1.8±0.2 | 1.6±0.2 | 1.6±0.2 | 2±0.2 | 1.6±0.2 | 0.0073 | 0.9590 | 0.3584 |
| Fibrinogen,  mg/dL | 337.5±18.0 | 363.9±17.0^c^ | 328.9±15.2 | 0.0056 | 341.2±25.7 | 365.0±24.1 | 344.4±21.6 | 333.7±26.0 | 363.8±24.5^c^ | 314.2±21.4 | 0.0052 | 0.6727 | 0.4349 |
| hs-CRP,  nmol/L | 17.1±12.4 | 24.8±11.4^c^ | 18.1±11.4 | 0.0029 | 20.9±13.3 | 26.7±12.4 | 20.9±12.4 | 14.3±13.3 | 21.9±12.4 | 16.2±12.4 | 0.0031 | 0.4873 | 0.8887 |
| *reported as geometric means ± SEM | | | | | | | | | | | | | |

**Table S2. Changes in lipoprotein subclasses for men and women during fasting.** Statistically significant differences between the three time points by means of adjusted p-values are indicated with ^a^: 0 d (baseline) vs. 7 d; ^b^: 0 d vs. 14 d and ^c^: 7 d vs. 14 d. The overall p-value is calculated by means of linear mixed models for repeated measures, with unstructured covariance structure to model within-subjects errors. Multiple comparison adjustment for heterogeneous variance between group was applied. Values are indicated as mean±SEM.

|  | **all** |  |  |  | **men** |  |  | **women** |  |  | **overall p-value** |  |  |
| --- | --- | --- | --- | --- | --- | --- | --- | --- | --- | --- | --- | --- | --- |
| **Parameter** | **0 d** | **7 d** | **14 d** | **p-value** | **0 d** | **7 d** | **14 d** | **0 d** | **7 d** | **14 d** | **time** | **sex** | **time* sex** |
| LDL1-C,  mmol/L | 1.00±0.07^b^ | 1.06±0.08^c^ | 0.84±0.08 | <0.0001 | 0.93±0.10 | 1.06±0.12^c^ | 0.78±0.11 | 1.07±0.10 | 1.05±0.12^c^ | 0.90±0.11 | <0.0001 | 0.5540 | 0.0618 |
| LDL2-C,  mmol/L | 1.31±0.08^b^ | 1.30±0.09^c^ | 1.02±0.09 | <0.0001 | 1.28±0.11^b^ | 1.34±0.13^c^ | 1.00±0.13 | 1.34±0.11^b^ | 1.27±0.13^c^ | 1.04±0.13 | <0.0001 | 0.9439 | 0.1362 |
| LDL3-C,  mmol/L | 0.89±0.06^b^ | 0.83±0.06^c^ | 0.63±0.05 | <0.0001 | 0.95±0.08^b^ | 0.82±0.09^c^ | 0.61±0.07 | 0.84±0.08^b^ | 0.84±0.09^c^ | 0.64±0.07 | <0.0001 | 0.8205 | 0.2282 |
| LDL1-TG,  mmol/L | 0.14±0.01^a,b^ | 0.21±0.01 | 0.2±0.01 | <0.0001 | 0.15±0.01^a,b^ | 0.22±0.01 | 0.21±0.01 | 0.14±0.01^a,b^ | 0.21±0.01 | 0.2±0.01 | <0.0001 | 0.3186 | 0.9060 |
| LDL2-TG,  mmol/L | 0.09±0^a,b^ | 0.14±0.01 | 0.13±0.01 | <0.0001 | 0.1±0.01^a,b^ | 0.14±0.01 | 0.14±0.01 | 0.09±0.01^a,b^ | 0.13±0.01 | 0.12±0.01 | <0.0001 | 0.2812 | 0.6852 |
| LDL3-TG,  mmol/L | 0.08±0^a,b^ | 0.11±0 | 0.1±0 | <0.0001 | 0.09±0.01 | 0.11±0.01 | 0.11±0.01 | 0.08±0.01^a,b^ | 0.11±0.01 | 0.1±0.01 | <0.0001 | 0.7294 | 0.1056 |
| HDL2-C,  mmol/L | 1.10±0.06^a,b^ | 1.01±0.05 | 1.00±0.06 | 0.0261 | 0.90±0.07 | 0.83±0.07 | 0.87±0.08 | 1.31±0.07^b^ | 1.19±0.07 | 1.12±0.08 | 0.0232 | 0.0006 | 0.0359 |
| HDL3-C,  mmol/L | 0.25±0.01^a,b^ | 0.21±0.01 | 0.19±0.01 | <0.0001 | 0.23±0.01^a,b^ | 0.19±0.01 | 0.18±0.01 | 0.14±0.03 | 0.15±0.03 | 0.14±0.03 | <0.0001 | 0.0065 | 0.8685 |
| HDL2-TG,  mmol/L | 0.13±0 | 0.13±0 | 0.13±0.01 | 0.2659 | 0.13±0.01 | 0.13±0.01 | 0.13±0.01 | 0.57±0.03^b^ | 0.52±0.03 | 0.49±0.03 | 0.2581 | 0.9741 | 0.3147 |
| HDL3-TG,  mmol/L | 0.07±0.01 | 0.07±0.01 | 0.07±0.01 | 0.3705 | 0.08±0.01 | 0.08±0.01 | 0.07±0.01 | 0.12±0.01^a,b^ | 0.1±0 | 0.09±0 | 0.3777 | 0.0091 | 0.4960 |

**Table S3. Changes in ratios, reflecting the lipid content within the lipoproteins, during fasting.** Statistically significant differences between the three time points by means of adjusted p-values are indicated with ^a^: 0 d (baseline) vs. 7 d; ^b^: 0 d vs. 14 d and ^c^: 7 d vs. 14 d. The overall p-value is calculated by means of linear mixed models for repeated measures, with unstructured covariance structure to model within-subjects errors. Multiple comparison adjustment for heterogeneous variance between group was applied. Values are indicated as mean±SEM.

|  | **all** |  |  |  | **men** |  |  | **women** |  |  | **overall p-value** |  |  |
| --- | --- | --- | --- | --- | --- | --- | --- | --- | --- | --- | --- | --- | --- |
| **Parameter** | **0 d** | **7 d** | **14 d** | **p-value** | **0 d** | **7 d** | **14 d** | **0 d** | **7 d** | **14 d** | **time** | **sex** | **time* sex** |
| LDL-C/HDL-C | 2.5±0.2^b^ | 2.8±0.2^c^ | 2.2±0.2 | <0.0001 | 2.9±0.2 | 3.2±0.3 | 2.4±0.3 | 2.1±0.2 | 2.4±0.3^c^ | 2.0±0.3 | <0.0001 | 0.0568 | 0.0011 |
| LDL-TG/  HDL-C | 0.1±0.0^a,b^ | 0.1±0.0^c^ | 0.2±0.0 | <0.0001 | 0.1±0.0^a,b^ | 0.1±0.0^c^ | 0.2±0.0 | 0.1±0.0^a,b^ | 0.1±0.0 | 0.2±0.0 | <0.0001 | 0.3941 | 0.1761 |
| LDL-TG/  HDL-TG | 1.6±0.1^a,b^ | 2.2±0.1 | 2.4±0.1 | <0.0001 | 1.6±0.1^a,b^ | 2.3±0.1 | 2.4±0.2 | 1.6±0.1^a,b^ | 2.2±0.1 | 2.3±0.2 | <0.0001 | 0.6592 | 0.8109 |
| HDL-TG/  HDL-C | 0.2±0.5^a^ | 0.2±0.5 | 0.2±0.5 | 0.0384 | 0.2±0.5 | 0.2±0.5 | 0.2±0.5 | 0.1±0.5 | 0.2±0.5 | 0.2±0.5 | 0.0394 | 0.0004 | 0.5352 |
| VLDL-C/ VLDL-TG | 0.5±2.5^a,b^ | 0.7±2.5 | 0.7±2.5 | <0.0001 | 0.5±2.5^a^ | 0.7±2.5 | 0.7±2.5 | 0.7±2.5 | 0.7±2.5 | 0.7±2.5 | 0.0001 | 0.7716 | 0.0674 |
| TG/HDL-C | 1.2±0.1 | 1±0 | 1±0 | 0.2 | 1.5±0.1 | 1.2±0.1 | 1.2±0.1 | 0.9±0.1 | 0.9±0.1 | 0.8±0.1 | 0.1945 | 0.0001 | 0.2570 |
| TC/HDL-C | 4.0±0.2 | 4.2±0.2^c^ | 3.7±0.2 | <0.0001 | 4.5±0.2 | 4.7±0.3^c^ | 4.0±0.3 | 3.4±0.2 | 3.7±0.3^c^ | 3.4±0.3 | <0.0001 | 0.0206 | 0.0340 |

**Table S4.** **Changes in lipoprotein size and particle concentration for men and women during fasting.** Statistically significant differences between the three time points by means of adjusted p-values are indicated with ^a^: 0 d (baseline) vs. 7 d; ^b^: 0 d vs. 14 d and ^c^: 7 d vs. 14 d. The overall p-value is calculated by means of linear mixed models for repeated measures, with unstructured covariance structure to model within-subjects errors. Multiple comparison adjustment for heterogeneous variance between group was applied. Values are indicated as mean±SEM.

|  | **all** |  |  |  | **men** |  |  | **women** |  |  | **overall p-value** |  |  |
| --- | --- | --- | --- | --- | --- | --- | --- | --- | --- | --- | --- | --- | --- |
| **Parameter** | **0 d** | **7 d** | **14 d** | **p-value** | **0 d** | **7 d** | **14 d** | **0 d** | **7 d** | **14 d** | **time** | **sex** | **time* sex** |
| VLDL-s, nm | 47.57±0.69 | 47.37±0.34 | 47.63±0.33 | 0.5181 | 48.79±0.94 | 46.40±0.44 | 46.77±0.43 | 46.35±0.94 | 48.34±0.44 | 48.48±0.43 | 0.5192 | 0.5623 | 0.004 |
| LDL-s, nm | 21.10±0.07 | 21.11±0.05 | 21.07±0.05 | 0.1657 | 20.88±0.09 | 21.05±0.07 | 21.02±0.07 | 21.31±0.09 | 21.17±0.07 | 21.12±0.07 | 0.1741 | 0.0221 | 0.0232 |
| HDL-s, nm | 9.07±0.09^a,b^ | 9.24±0.08^c^ | 9.39±0.07 | <0.0001 | 8.75±0.10^b^ | 8.95±0.10^c^ | 9.19±0.09 | 9.39±0.10 | 9.52±0.10 | 9.58±0.09 | <0.0001 | 0.0002 | 0.005 |
| Large VLDL-p,  nmol/L | 4.74±1.15^a,b^ | 1.94±1.08 | 1.90±1.04 | <0.0001 | 5.59±1.19^a,b^ | 1.82±1.11 | 1.91±1.05 | 3.60±1.23^b^ | 2.08±1.12 | 1.89±1.07 | <0.0001 | 0.3659 | 0.2124 |
| LDL-p,  nmol/L | 1479.33± 73.92^b^ | 1477.73± 83.47^c^ | 1186.16± 76.80 | <0.0001 | 1574.25± 103.64^b^ | 1558.35± 118.15^c^ | 1196.95± 110.11 | 1384.40± 103.64 | 1397.10± 118.15 ^c^ | 1182.72± 110.87 | <0.0001 | 0.419 | 0.0548 |
| Large LDL-p,  nmol/L | 889.28± 53.14^b^ | 822.98± 57.42^c^ | 630.37± 54.24 | <0.0001 | 821.40± 74.52^b^ | 807.50± 82.18^c^ | 597.80± 76.96 | 957.15± 74.52^b^ | 838.45± 82.18 ^c^ | 665.11± 77.33 | <0.0001 | 0.4584 | 0.1524 |
| Small LDL-p,  nmol/L | 589.95± 48.33 | 654.78± 42.00^c^ | 555.16± 34.84 | 0.0001 | 752.85± 58.28 | 750.85± 55.99^c^ | 599.10± 48.87 | 427.05± 58.28 | 558.70± 55.99 | 513.99± 49.49 | 0.0001 | 0.0047 | 0.0033 |
| HDL-p,  nmol/L | 36240.98± 1156.49^a,b^ | 30920.88± 663.18^c^ | 28312.77± 613.13 | <0.0001 | 35920.40± 1655.27^a,b^ | 29954.70± 923.92^c^ | 27013.45± 823.18 | 36561.55± 1655.27 ^a,b^ | 31887.05± 923.92^c^ | 29637.69± 829.92 | <0.0001 | 0.2193 | 0.5681 |
| Large HDL-p,  nmol/L | 6703.37± 699.07 | 6929.07± 666.88 | 7507.72± 561.84 | 0.0419 | 4236.70± 826.38 | 4630.50± 796.74^c^ | 5865.80± 711.58 | 9170.05± 826.38 | 9227.65± 796.74 | 9131.48± 714.30 | 0.0227 | 0.0001 | 0.0084 |
| Small HDL-p,  nmol/L | 29537.60± 1123.55^a,b^ | 23991.80± 758.13^c^ | 20792.83± 563.74 | <0.0001 | 31683.75± 1532.56^a,b^ | 25324.20± 1042.28^c^ | 21147.65± 802.41 | 27391.45± 1532.56^a,b^ | 22659.40± 1042.28^c^ | 20469.12± 809.35 | <0.0001 | 0.0908 | 0.0526 |


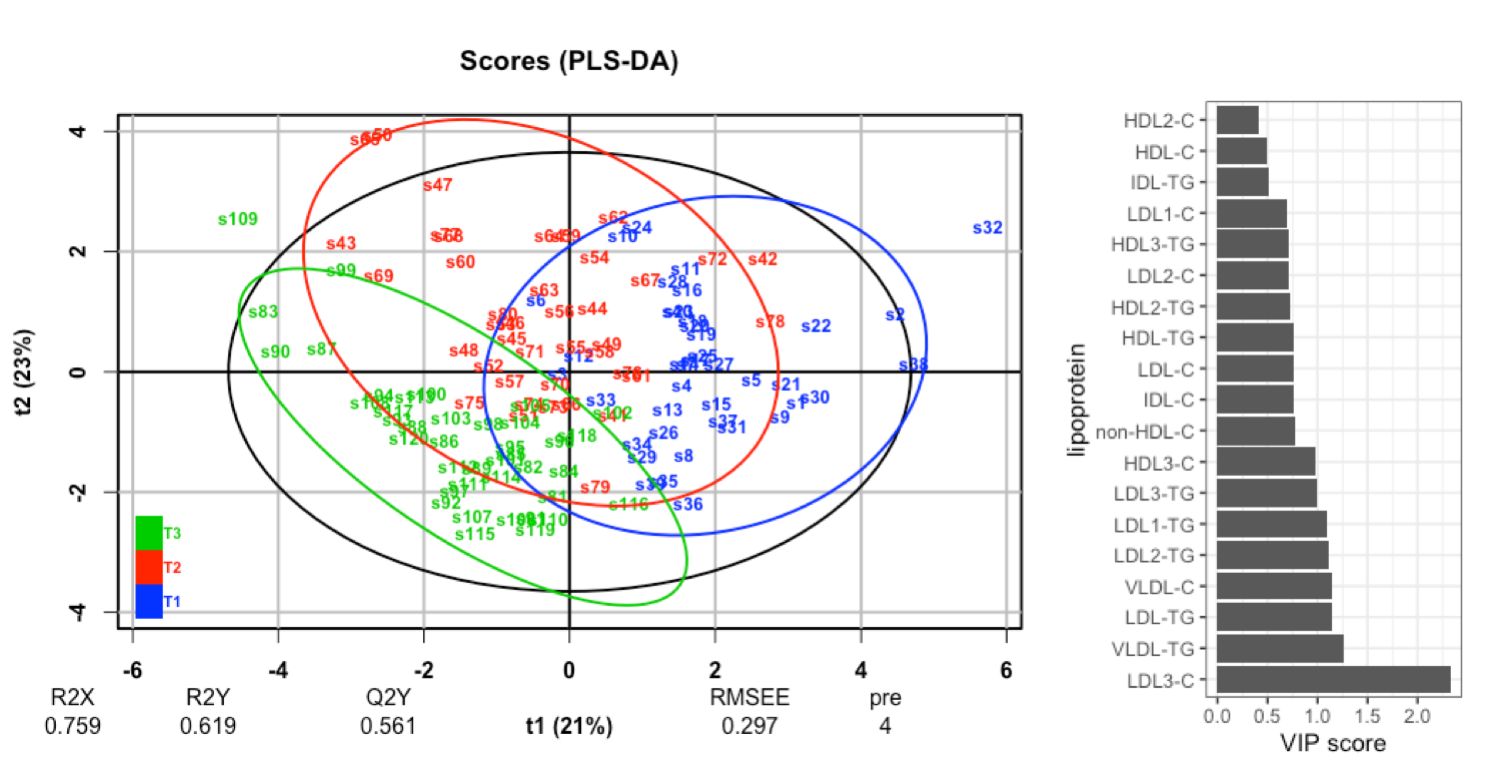


**Fig. S1. PLS-DA of lipoprotein subclasses separated by density gradient ultracentrifugation.** Sample clustering (left panel) shows that most of the variation between the different groups is attributable to fasting. Variable Importance in Projection (VIP) scores (right panel) show the identity of lipoproteins most significantly explaining the differences between the three groups. R2X, R2Y: fraction of the variance of predictors (X) and response (Y). Q2Y, predictive performance of the model by cross-validation. RMSEE, Root Mean Squared Error of Estimation. VIP score, variable importance in projection score.

**Table S5. Inter- and intra-assay coefficient of variation (CV) for lipoprotein(a) measurements.**

| **inter-assay** | **mean (nmol/L)** | **SD (nmol/L)** | **CV %** |
| --- | --- | --- | --- |
| level 1 | 10.6 | 3.6 | 34.5% |
| level 2 | 72.0 | 1.7 | 2.4% |
| level 3 | 288.5 | 3.1 | 1.1% |
|  |  |  |  |
| **intra-assay** | **mean (nmol/L)** | **SD (nmol/L)** | **CV %** |
| level 1 | 9.9 | 0.4 | 3.8% |
| level 2 | 64.7 | 0.8 | 1.3% |
| level 3 | 264.0 | 10.5 | 3.2% |
